# Impact of COVID-19 Related Knowledge and Precautions on Emotional and Behavioral Problems Among Children During the Post-pandemic in China: The Explanatory Value of Emotional Problems Among Caregivers

**DOI:** 10.3389/fpsyg.2021.712529

**Published:** 2021-10-13

**Authors:** Jingyi Wang, Yun Chen, Xiaoqin Guo, Haijiang Lin, Marcus Richards, Hao Wang, Xiaoxiao Chen, Chaowei Fu

**Affiliations:** ^1^Key Laboratory of Public Health Safety, NHC Key Laboratory of Health Technology Assessment, School of Public Health, Fudan University, Shanghai, China; ^2^Songjiang Center for Disease Control and Prevention, Shanghai, China; ^3^Taizhou City Center for Disease Control and Prevention, Taizhou, China; ^4^MRC Unit for Lifelong Health and Ageing, Institute of Cardiovascular Science, University College London, London, United Kingdom

**Keywords:** COVID-19, knowledge, precaution, emotional and behavioral problems, children, caregivers

## Abstract

To investigate the prevalence of emotional and behavioral problems (EBPs) among children during the COVID-19 post-pandemic in China; examine associations between COVID-19-related knowledge and precautions and problems in children, and explore the potential explanatory value of the mental health status of caregivers on any associations observed. Based on a cross-sectional design, caregivers of 6,017 children from 12 primary schools in Shanghai and Taizhou, China, were invited to complete an online survey from June 26 to July 6, 2020. EBPs of the children were assessed using the Strengths and Difficulties Questionnaire (SDQ), while the emotional problems of caregivers were assessed using the Depression Anxiety Stress Scales-21 (DASS-21). Structural equation modeling was employed to estimate the direct and indirect associations (explained by the emotional problems of caregivers) between COVID-19-related knowledge and precautions and the EBPs among children. The overall prevalence of EBPs in the sample was 12.5%, and 5.3% of them had a high or very high SDQ total difficulties score during the COVID-19 post-pandemic. After adjustment for covariates, higher COVID-19-related knowledge (β = −0.83; *P* < 0.001) and precautions (β = −0.80; *P* < 0.001) were significantly associated with lower SDQ total difficulties score among children. There was an explanatory effect of emotional problems of caregivers on the aforementioned associations, which explained 31% and 41% of the total effect, respectively. Higher levels of knowledge and precautions of COVID-19 were associated with lower EBPs among children, and the relationship was partially explained by the emotional problems in caregivers. It may be beneficial to improve pandemic-related prevention education and adopt psychological interventions toward the emotional status of caregivers for the psychological health of children.

## Introduction

COVID-19, the infectious disease caused by the novel coronavirus SARS-COV-2, was first identified in Wuhan, China in December 2019 (Zhu et al., 2020), and has rapidly become the worst global health pandemic in a century. In response to the outbreak, most of the countries have made unprecedented efforts to practice “social distancing” (Shen et al., 2020). As a result, mass gatherings and public events were banned; schools, public buildings, and businesses were closed; and travel was restricted (Chen et al., 2020; Van Lancker and Parolin, 2020). Global risk of depression and anxiety was caused by the detrimental effect of these measures, including home quarantine, economic recession, negative information overload, and fear of infection (Agha, 2020). The pandemic not only exacerbated existing mental health problems but also led to more clinical psychiatric cases among the general population (Golberstein et al., 2020). Children are particularly vulnerable owing to their limited understanding of the event, and may not be able to avoid physical and mental problems due to their limited coping strategies (Dalton et al., 2020). Furthermore, because of the closure of schools, children experienced a prolonged state of home isolation and lacked healthcare knowledge, in-person contact with peers and teachers, and personal space, which could hurt their mental health (Brooks et al., 2020; Van Lancker and Parolin, 2020). Although the education sector had launched online education to minimize the learning loss, there were still a substantial proportion of students who were not satisfied with online education and were not participating effectively, especially those from families or regions with lower socioeconomic status (Ma et al., 2021). The problems related to studies, such as having difficulty in studying at home and dislike of remote learning, have been reported to be prevalent among adolescents during the closure of schools, and they were associated with more severe depressive symptoms (Wang et al., 2021). Among hospitals that provide mental health services to children and adolescents in China, 9% closed their outpatient units and 25% closed inpatient wards during the COVID-19 outbreak (Cui et al., 2020). Although over 15% of these hospitals launched online services, the disruption of previous mental health services had a negative influence on children and their caregivers (Cui et al., 2020).

Previous studies have reported the prevalence of mental health problems in children during the pandemic period (Duan et al., 2020; Jiao et al., 2020; University of Oxford, 2020; Xie et al., 2020). A study in Hubei province found that 22.6 and 18.9% of 2,330 primary school students suffered from depression and anxiety, respectively (Xie et al., 2020). Similarly, another study investigated 359 children and 3,252 adolescents by online survey and revealed that the prevalence of depressive symptoms in children and adolescents was 22.28% during the COVID-19 outbreak (Duan et al., 2020). Many children aged 3–18 years in China displayed psychological problems including clinginess, inattention, irritability, and fear of asking questions about the pandemic (Jiao et al., 2020). An online survey conducted among over 1,500 parents by the CO-SPACE study in the UK suggested that children had a high level of COVID-19-related worries and fears, with the number of younger children (aged 4–10 years) being significantly higher than older children (aged 11–16 years) (University of Oxford, 2020).

At the same time, caregivers of children were also at high risk of mental health problems owing to the fear of infection in family members, economic problems in the family, and educational attainment of children (Spinelli et al., 2020; Wang et al., 2020a). It also resulted in stress for caregivers as they had to inform and explain to their children about the pandemic and manage their fears (Fegert et al., 2020). Previous studies suggest an increased risk of EBPs among children whose parents had depression (Psychogiou et al., 2017). The pressure that caregivers suffered could lessen their parenting abilities and convey anxiety to their children (Heinrich, 2014). A cross-sectional study conducted in Bangladesh, during the pandemic, reported that children of parents who needed to go to the workplace or were at risk of losing their jobs tended to have a higher level of stress and increased mental disorders (Yeasmin et al., 2020).

As COVID-19 is a new disease and has devastating effects globally, knowledge and compliance with coronavirus precautions are crucial to prevent and alleviate mental health problems during the pandemic. Studies of public health crises suggest that adequate knowledge during the epidemic largely influenced the degree of adopting preventive behaviors among the general population and ultimately protected mental health (Brug et al., 2004; Sadique et al., 2007; Yildirim et al., 2020). The importance of self-perceived knowledge has also been emphasized by existing studies. Self-perceived knowledge, which moderately correlated with actual knowledge (Flynn and Goldsmith, 1999; Krawczyk et al., 2013), increased the confidence in a person's knowledge which could translate into more information search, perceived benefits of adopting preventative behaviors, and all phases of the decision-making process (Park et al., 1988; Krawczyk et al., 2013). With the widespread use of social media, conspiracy theories about the pandemic spread rapidly, which often disturbed emotional well-being (Desta and Mulugeta, 2020). Having poor knowledge and preventive behaviors owing to erroneous media information and pandemic rumors could harm the mental health of children (Rosling and Rosling, 2003). Recent studies suggest that a lack of knowledge about the pandemic and inadequate preventive behaviors affected the overall mental health (Yildirim and Güler, 2020), and increased depression, stress, and anxiety (Du et al., 2020; Wang et al., 2020b).

Although some previous studies reported the prevalence of psychological problems among children or their caregivers during the pandemic, very little is known about the prevalence during the post-pandemic period in China when the epidemic was under control, and about the associations between knowledge of and preventive behaviors against COVID-19, the mental health status of caregivers, and psychopathology of children. Therefore, we conducted a cross-sectional study to assess the EBPs among children when the schools reopened. We also aimed to investigate the association between COVID-19-related knowledge and preventive behaviors (precautions) and the EBPs in children, and also explore whether the emotional problems in caregivers would explain the association.

## Materials and Methods

### Participants and Design

A cross-sectional study was conducted among primary school children and their caregivers in Taizhou and Shanghai, China, from June 26 to July 6, 2020, when local schools had fully reopened. Cluster sampling was adopted, and six primary schools each were randomly sampled from Taizhou and Shanghai, respectively, which included public and non-public schools. Three classes were randomly sampled from each grade (first to fifth grade) in each school. All the caregivers of children in the chosen classes were invited to complete an online Chinese questionnaire through the Wenjuanxing platform (https://www.wjx.cn), with an average response time of 18.6 ± 10.7 min. Eligibility criteria were: (1) caregivers of children in first to fifth grade; (2) should be able to read, understand, and complete the survey questionnaire independently; and (3) children and their caregivers should provide online informed consent. After excluding 34 invalid questionnaires and 349 with missing data on school information, 6,017 participants were included for the current analyses. This study was approved by the Institutional Review Board of the School of Public Health, Fudan University, Shanghai, China (IRB#2020040817), and all participants provided online informed consent.

### Assessment of Sociodemographic Characteristics

Demographic information included children's age, gender (male or female), type of school (key school or not), grade (first to fifth), family economic status (low, medium, and high), and educational attainment of caregivers (primary school or lower, middle school, high school, and college or higher).

### Assessment of COVID-19 Pandemic Characteristics

Knowledge and precaution levels of caregivers and children regarding COVID-19 were evaluated based on four items: Q1. How much do you know about COVID-19-related knowledge? Q2. How much do you think your child knows about COVID-19-related knowledge? Q3. How many precautions do you take to prevent the spread of COVID-19? Q4. How many precautions do you think your child takes to prevent the spread of COVID-19? Each item consisted of seven options, scored from 1 (Very poor knowledge/Not at all) to 7 (Very good knowledge/Completely following the recommendations from authorities), and the items were treated as continuous variables, with a higher score indicating a better understanding and prevention of the infectious disease. The COVID-19 knowledge and precaution questionnaire showed good internal consistency (Cronbach's alpha was 0.79).

### Assessment of Emotional and Behavioral Problems in Children

The EBPs in children were measured by the parent's version of the Strengths and Difficulties Questionnaire (SDQ) (Goodman, 1997). The SDQ is a 25-item measure that asks caregivers to rate their child's behavior over the past 6 months, using five subscales—emotional problems, conduct problems, hyperactivity, peer problems, and prosocial behavior in young people aged 4–17 years. Each scale includes five items and each item consists of four statements scored from 0 to 2 (ranging from “did not apply to me at all” = 0; “applied to me some of the time” = 1; and “applied to me very much or most of the time” = 2). Caregivers were asked to choose the best statement that described their child's feelings and thoughts. The total difficulties score was generated by summing the scores from all the scales except prosocial. The resultant score ranged from 0 to 40, with higher scores indicating more EBPs. A 4-fold classification was used to divide different groups based on the scores: 0–13 indicated close to average, 14–16 indicated slightly high, 17–19 indicated high, and 20 to 40 indicated very high. The Chinese version of the SDQ showed good reliability and validity (Cronbach's alpha for the total difficulties score = 0.86) (Liu et al., 2013).

### Assessment of the Emotional Problems of Caregivers

In this study, 99.4% of caregivers of children were their parents, and 0.6% were step-parents or grandparents. The Depression Anxiety Stress Scales-21 (DASS-21) was used to measure the states of depression, anxiety, and stress in these caregivers (Henry and Crawford, 2005). The DASS-21 is a 21-item self-reported scale, which asks caregivers to respond to each item by rating the best statement applied over the past week, using a score from 0 to 3 (ranging from “did not apply to me at all” = 0; “applied to me some of the time” = 1; “applied to me a good part of the time” = 2; and “applied to me very much or most of the time” = 3). Each subscale of depression, anxiety, and stress ranged from 0 to 21, with higher scores indicating greater severity of symptoms. The Cronbach's alpha of DASS-21 for depression anxiety, and stress was 0.90, 0.82, and 0.87 in the previous study (Lee, 2019), and 0.81, 0.79, and 0.81, respectively, in this study.

### Statistical Analysis

Continuous data were described as means (SD) and categorical data as frequencies and percentages. Chi-square tests and one-way ANOVA were used to compare the categorical and continuous variables among different SDQ groups. Pearson's correlation analysis tested the relation between these variables. Two structural equation models (SEMs) were used to estimate whether associations between COVID-19-related knowledge or precautions and children's total difficulties score were explained by the DASS-21 score of caregivers. COVID-19-related knowledge was represented as a latent variable based on the knowledge of caregivers and children about the disease. COVID-19 related precautions were represented as a latent variable based on the precautions of caregivers and children about the disease. The DASS-21 score of caregivers was represented as a latent variable based on the three subscales of depression, anxiety, and stress. The models were adjusted for gender, grade, school type, family income status, and educational attainment of caregivers. We first tested the SEM model built on a pre-specified theoretical assumption. Subsequently, we re-specified the model by removing non-significant associations and re-evaluating its fitness. Effect sizes of the explanatory factor were calculated as the indirect effect divided by the total effect, according to MacKinnon's formula (Mackinnon, 2008). Chi-square test, root means square error of approximation (RMSEA), the comparative fit index (CFI), Tucker Lewis index (TLI), and standardized root mean square residual (SRMR) were used to test how well the models fitted the observed data, where an RMSEA and SRMR value < 0.05 and CFI and TLI value more than 0.95 indicated an adequate model fit. The regression coefficient was used to quantify the strength of the relationship between pairs of variables in the SEM model, and bootstrapping for 10,000 times was used to estimate the SE. A sensitivity analysis was performed in which COVID-19-related knowledge and precautions of caregivers and children were treated as independent variables. This was done to examine whether the latent variable models were misspecified and to determine the robustness of the primary analyses. All analyses were performed by *Mplus* (Muthén and Muthén, 1998) and *R* (version 4.0.1; R Foundation for Statistical Computing, Vienna, Austria). All statistical tests were two-sided and the level of significance was set at a *P* < 0.05.

## Results

### Characteristics of the Study Population

Table 1 presents descriptive information on the study population. Of the 6,017 participants, 54.6% were boys. The mean (SD) SDQ total difficulties score of children was 8.1 (4.5) in this study. The prevalence of slightly high, high, and very high EBPs was 7.2, 3.3, and 2.0%, respectively. Boys and children of caregivers with lower educational attainment and family economic status had a higher prevalence of EBPs. The mean COVID-19-related knowledge scores of caregivers and children were 5.3 ± 1.7 (min: 1.0, max: 7.0) and 4.5 ± 1.8 (min: 1.0, max: 7.0), respectively, and the mean COVID-19-related precautions scores were 6.5 ± 1.1 (min: 1.0, max: 7.0) for both. Compared to children without EBPs, children with those problems and their caregivers rated lower for COVID-19-related knowledge and precautions. Caregivers of children with EBPs had higher depression, anxiety, and stress scores. The distributions of school type and grade were not different across the levels of SDQ score.

**Table 1:** Characteristics of participants.

**Characteristics**	**Total**	**Strengths & Difficulties Questionnaire (SDQ) total difficulties score level**				***P*-value**
		**Close to average (0–13)**	**Slightly raised (14–16)**	**High (17–19)**	**Very high (20–40)**	
*n*	6,017	5,267 (87.5)	431 (7.2)	201 (3.3)	118 (2.0)	
SDQ total difficulties score (range 0–40)	8.1 (4.5)	6.8 (3.2)	14.8 (0.8)	17.8 (0.8)	21.7 (2.3)	<0.001
**Sociodemographic variables**						
Gender, *n* (%)						<0.001
Male	3,287 (54.6)	2,810 (53.4)	267 (61.9)	130 (64.7)	80 (67.8)	
Female	2,730 (45.4)	2,457 (46.6)	164 (38.1)	71 (35.3)	38 (32.2)	
School, *n* (%)						0.506
Key school	1,092 (18.2)	971 (18.4)	68 (15.8)	33 (16.4)	21 (17.8)	
Non-key school	4,924 (81.8)	42,96 (81.6)	363 (84.2)	168 (83.6)	97 (82.2)	
Grade, *n* (%)						0.265
First grade	1,269 (21.1)	1,127 (21.4)	91 (21.1)	35 (17.4)	16 (13.6)	
Second grade	1,200 (19.9)	1,045 (19.8)	87 (20.2)	37 (18.4)	31 (26.3)	
Third grade	1,262 (21.0)	1,086 (20.6)	104 (24.1)	44 (21.9)	28 (23.7)	
Fourth grade	1,233 (20.5)	1,077 (20.4)	85 (19.7)	50 (24.9)	21 (17.8)	
Fifth grade	1,053 (17.5)	932 (17.7)	64 (14.8)	35 (17.4)	22 (18.6)	
Caregivers' Education, *n* (%)						<0.001
Primary school or lower	415 (6.9)	331 (6.3)	51 (11.8)	15 (7.5)	18 (15.3)	
Middle school	1,750 (29.1)	1,506 (28.6)	150 (34.8)	56 (27.9)	38 (32.2)	
High school	1,500 (24.9)	1,302 (24.7)	110 (25.5)	64 (31.8)	24 (20.3)	
College or higher	2,352 (39.1)	2,128 (40.4)	120 (27.8)	66 (32.8)	38 (32.2)	
Economic status, *n* (%)						<0.001
High	620 (10.3)	569 (10.8)	31 (7.2)	12 (6.0)	8 (6.8)	
Middle	4,927 (81.9)	4,346 (82.5)	344 (79.8)	152 (75.6)	85 (72.0)	
Low	470 (7.8)	352 (6.7)	56 (13.0)	37 (18.4)	25 (21.2)	
**COVID-19 related knowledge**						
Caregivers (range 1–7)	5.3 (1.7)	5.4 (1.7)	4.7 (1.8)	4.6 (1.9)	4.6 (1.9)	<0.001
Children (range 1–7)	4.5 (1.8)	4.6 (1.8)	3.9 (1.9)	3.8 (1.8)	3.8 (1.9)	<0.001
**COVID-19 related precautions**						
Caregivers (range 1–7)	6.5 (1.1)	6.5 (1.1)	6.3 (1.3)	6.0 (1.6)	6.0 (1.6)	<0.001
Children (range 1–7)	6.5 (1.1)	6.5 (1.1)	6.2 (1.4)	6.0 (1.6)	5.9 (1.6)	<0.001
**Caregivers' DASS-21 score**						
Depression (range 0–21)	2.0 (2.0)	1.7 (1.6)	3.2 (2.6)	4.4 (3.4)	5.6 (4.1)	<0.001
Anxiety (range 0–21)	1.9 (1.7)	1.6 (1.3)	2.9 (2.3)	4.1 (3.1)	4.9 (3.8)	<0.001
Stress (range 0–21)	2.9 (2.5)	2.5 (2.1)	4.7 (3.1)	6.0 (3.7)	6.8 (4.3)	<0.001

*Data are mean (SD) for continuous variables or n (%) for categorical variables. Chi-square test and one-way ANOVA were used to compare the categorical and continuous variables among different SDQ groups. SDQ, Strengths and Difficulties Questionnaire; DASS-21, Depression Anxiety Stress Scales-21*.

### COVID-19-Related Knowledge and Precautions, DASS-21 Score of Caregivers, and SDQ Total Difficulties Score of Children

**Figure 1:**
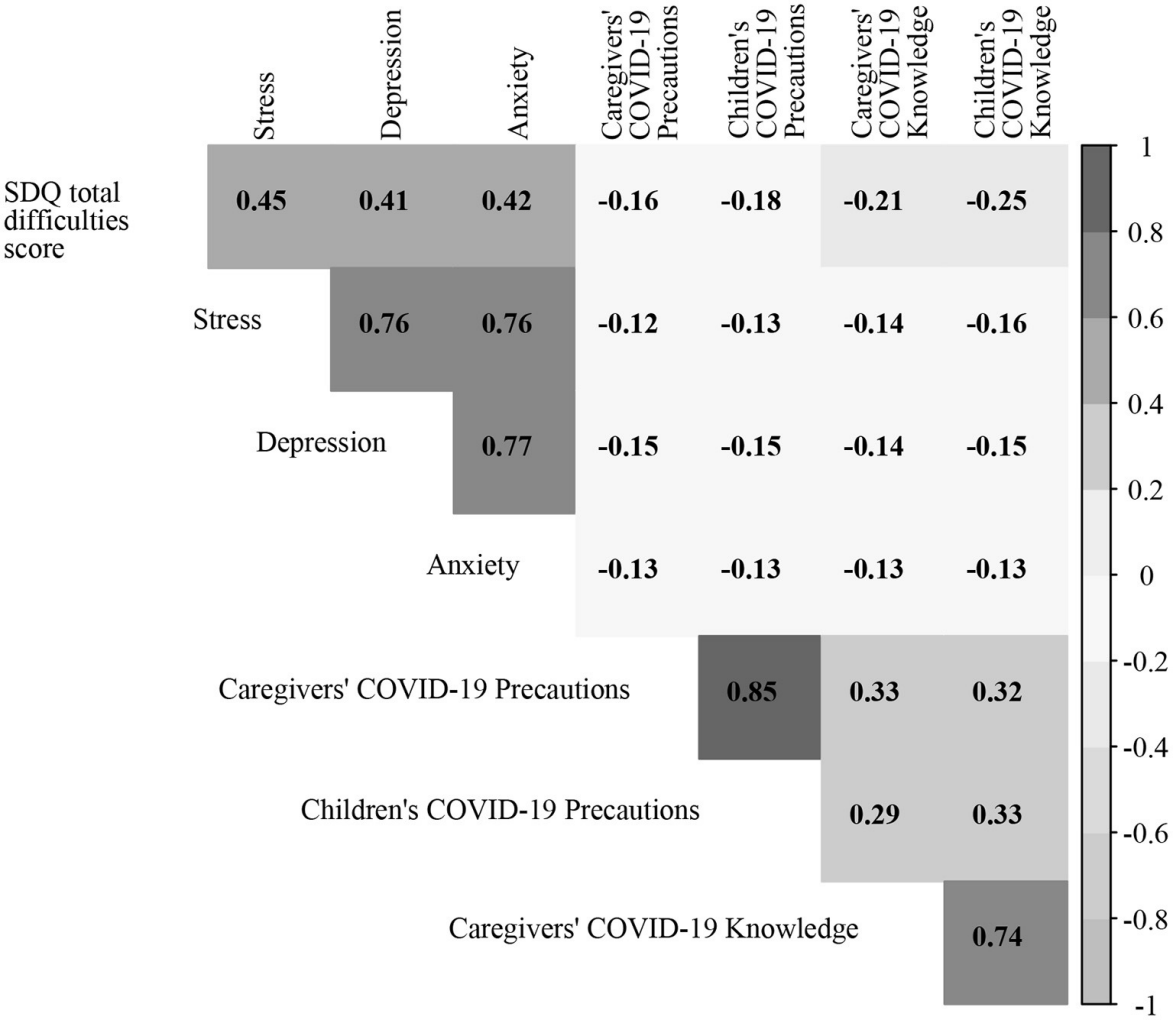
Plot of the correlation coefficient. All *P* < 0.05. SDQ, Strengths and Difficulties Questionnaire.

The correlation matrix for the indicator variables used in the models is presented in Figure 1. The total difficulties score of children was positively correlated with depression (*r* = 0.45, *P* < 0.001), anxiety (*r* = 0.41, *P* < 0.001), and stress of caregivers (*r* = 0.42, *P* < 0.001), while the score was negatively correlated with COVID-19-related knowledge (*r* = −0.25, *P* < 0.001) and precautions (*r* = −0.18, *P* < 0.001) of children as well as the COVID-19-related knowledge (*r* = −0.21, *P* < 0.001) and precautions (*r* = −0.16, *P* < 0.001) of their caregivers. Stress, depression, and anxiety of caregivers were positively inter-correlated and were negatively correlated, respectively, with the COVID-19-related knowledge and precautions of themselves and their children. COVID-19-related precautions among caregivers had a strong positive correlation with those of their children, while COVID-19-related knowledge among caregivers had a strong positive correlation with that of their children.

**Figure 2:**
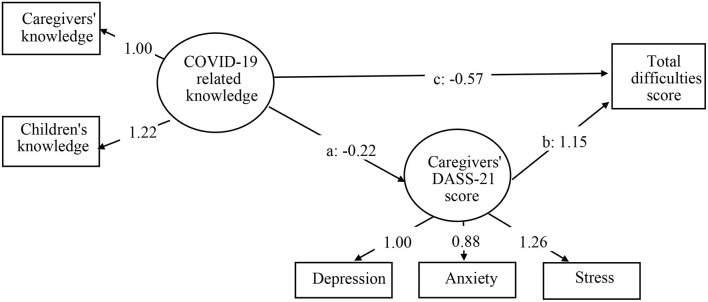
Model 1 of associations between COVID-19-related knowledge, DASS-21 score of caregivers, and total difficulties score. Data shown are unstandardized coefficients and all the associated *P* < 0.001. Model 1 adjusted for gender, grade, school, family economic status, and education level of caregivers. DASS-21, Depression Anxiety Stress Scales-21.

Figure 2 displays model 1 of the relationship between COVID-19-related knowledge and total difficulties score of children, which was explained by the depression, anxiety, and stress in caregivers. Model 1 adjusted for gender, grade, school type, family economic status, and educational attainment of caregivers. The model fit indices were acceptable with χ^2^ 927.198 (*df* = 71, *P* < 0.001), CFI 0.956, TLI 0.950, RMSEA 0.045, and SRMR 0.035. COVID-19-related knowledge was treated as a latent variable with two indicators, including the knowledge of caregivers and children (β = 1.22, *P* < 0.001). The DASS-21 score of caregivers was treated as a latent variable with three indicators, including depression, anxiety (β = 0.88, *P* < 0.001), and stress scales (β = 1.26, *P* < 0.001). The three path coefficients were all significant: path a from COVID-19-related knowledge to DASS-21 score of caregivers (β = −0.22, *P* < 0.001), path b from DASS-21 score of caregivers to total difficulties score of children (β = 1.15, *P* < 0.001), and the direct path c from COVID-19-related knowledge to total difficulties score of children (β = −0.57, *P* < 0.001). The bootstrapping index for an indirect effect (a ^*^ b: β = −0.26, *P* < 0.001) was also statistically significant with effect size 0.31, suggesting that the DASS-21 score of caregivers partially explained the relationship between COVID-19-related knowledge and total difficulties score of children.

**Figure 3:**
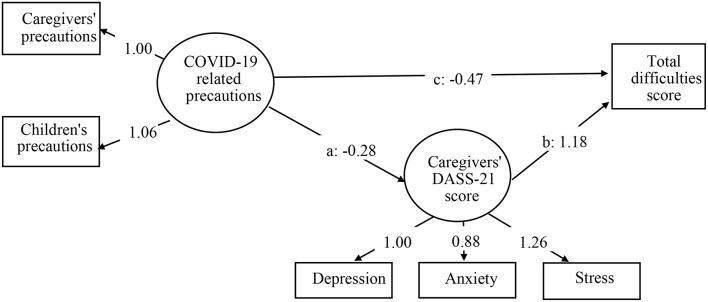
Model 2 of associations between COVID-19-related precautions, DASS-21 score of caregivers, and total difficulties score. Data shown are unstandardized coefficients and all the associated *P* < 0.001. Model 2 adjusted for gender, grade, school, family economic status, and education level of caregivers. DASS-21, Depression Anxiety Stress Scales-21.

Figure 3 displays model 2 of the relationship between COVID-19-related precautions and total difficulties score, which was explained by depression, anxiety, and stress in caregivers. Model 2 adjusted for the same covariates as model 1. The model fit indices were acceptable with χ^2^ 591.726 (*df* = 71, *P* < 0.001), CFI 0.976, TLI 0.973, RMSEA 0.035, and SRMR 0.034. COVID-19-related precautions were treated as a latent variable with two indicators, including precautions of caregivers and children (β = 1.06, *P* < 0.001). The three path coefficients were all significant: path a from COVID-19-related precautions to DASS-21 score of caregivers (β = −0.28, *P* < 0.001), path b from DASS-21 score of caregivers to total difficulties score of children (β = 1.18, *P* < 0.001), and the direct path c from COVID-19-related precautions to total difficulties score of children (β = −0.47, *P* < 0.001). The bootstrapping index for an indirect effect (a ^*^ b: β = −0.33, *P* < 0.001) was also statistically significant with effect size 0.41, suggesting that the DASS-21 score of caregivers partially explained the relationship between COVID-19-related precautions and total difficulties score of children. The bootstrapping results of the two models are summarized in Table 2.

**Table 2:** Statistical results of the multivariable models.

	**β**	***SE***	***b***	***P*-value**	**Effect size**
**Model 1**					
COVID-19 related knowledge → Caregivers' DASS-21 score (path a)	−0.22	0.02	−0.18	<0.001	
Caregivers' DASS-21 score → Total difficulties score (path b)	1.15	0.05	0.45	<0.001	
COVID-19 related knowledge → Total difficulties score (path c)	−0.57	0.04	−0.18	<0.001	
Indirect effect *via* Caregivers' DASS-21 score (a * b)	−0.26	0.02	–	<0.001	
Total effect of COVID-19 related knowledge on total difficulties score (a * b + c)	−0.83	0.07	–	<0.001	0.31
DASS-21					
Anxiety	0.88	0.02	0.88	<0.001	
Stress	1.26	0.03	0.87	<0.001	
**COVID-19 related knowledge**					
Children' knowledge	1.22	0.05	0.91	<0.001	
**Model 2**					
COVID-19 related precautions → Caregivers' DASS-21 score (path a)	−0.28	0.03	−0.16	<0.001	
Caregivers' DASS-21 score → Total difficulties score (path b)	1.18	0.05	0.46	<0.001	
COVID-19 related precautions → Total difficulties score (path c)	−0.47	0.06	−0.10	<0.001	
Indirect effect *via* Caregivers' DASS-21 score (a * b)	−0.33	0.04	–	<0.001	
Total effect of COVID-19 related precautions on total difficulties score (a * b + c)	−0.80	0.07	–	<0.001	0.41
DASS-21					
Anxiety	0.88	0.02	0.88	<0.001	
Stress	1.26	0.03	0.87	<0.001	
**COVID-19 related precautions**					
Children' precautions	1.06	0.06	0.94	<0.001	

*β = unstandardized estimate; b = standardized estimate. Effect size is the proportion mediated, which is calculated by dividing the indirect effect by the total effect. Models adjusted for gender, grade, school, family economic status, and education level of caregivers. DASS-21, Anxiety Stress Scales-21*.

### Sensitivity Analysis

A sensitivity analysis was performed in which COVID-19-related knowledge and precautions of caregivers and children were treated as independent variables. All the model fit indices were acceptable, and the bootstrapping results of the four models are summarized in Table 3. The results also revealed that COVID-19-related knowledge and precautions of caregivers and children were negatively associated with the total difficulties score, partially explained by the DASS-21 score of caregivers.

**Table 3:** Statistical results of the multivariable models for sensitivity analysis.

	**β**	***SE***	***b***	***P*-value**	**Effect size**
**Model 1**					
Caregivers' COVID-19 related knowledge → Caregivers' DASS-21 score (path a)	−0.15	0.02	−0.15	<0.001	
Caregivers' DASS-21 score → Total difficulties score (path b)	1.17	0.05	0.45	<0.001	
Caregivers' COVID-19 related knowledge → Total difficulties score (path c)	−0.36	0.03	−0.13	<0.001	
Indirect effect *via* caregivers' DASS-21 score (a * b)	−0.18	0.02	–	<0.001	
Total effect of caregivers' COVID-19 related knowledge on total difficulties score (a * b + c)	−0.54	0.07	–	<0.001	0.33
DASS-21					
Anxiety	0.88	0.02	0.88	<0.001	
Stress	1.26	0.03	0.87	<0.001	
**Model 2**					
Children' COVID-19 related knowledge → Caregivers' DASS-21 score (path a)	−0.15	0.01	−0.16	<0.001	
Caregivers' DASS-21 score → Total difficulties score (path b)	1.16	0.05	0.45	<0.001	
Children' COVID-19 related knowledge → Total difficulties score (path c)	−0.39	0.03	−0.16	<0.001	
Indirect effect *via* caregivers' DASS-21 score (a * b)	−0.17	0.02	–	<0.001	
Total effect of children' COVID-19 related knowledge on total difficulties score (a * b + c)	−0.57	0.03	–	<0.001	0.30
DASS-21					
Anxiety	0.88	0.02	0.88	<0.001	
Stress	1.26	0.03	0.87	<0.001	
**Model 3**					
Caregivers' COVID-19 related precautions → Caregivers' DASS-21 score (path a)	−0.24	0.03	−0.15	<0.001	
Caregivers' DASS-21 score → Total difficulties score (path b)	1.19	0.05	0.46	<0.001	
Caregivers' COVID-19 related precautions → Total difficulties score (path c)	−0.36	0.05	−0.09	<0.001	
Indirect effect *via* caregivers' DASS-21 score (a * b)	−0.28	0.03	–	<0.001	
Total effect of caregivers' COVID-19 related precautions on total difficulties score (a * b + c)	−0.64	0.06	–	<0.001	0.44
DASS-21					
Anxiety	0.88	0.02	0.88	<0.001	
Stress	1.26	0.03	0.87	<0.001	
**Model 4**					
Children' COVID-19 related precautions → Caregivers' DASS-21 score (path a)	−0.23	0.03	−0.15	<0.001	
Caregivers' DASS-21 score → Total difficulties score (path b)	1.18	0.05	0.46	<0.001	
Children' COVID-19 related precautions → Total difficulties score (path c)	−0.40	0.05	−0.10	<0.001	
Indirect effect *via* caregivers' DASS-21 score (a * b)	−0.27	0.03	–	<0.001	
Total effect of children' COVID-19 related precautions on total difficulties score (a * b + c)	−0.67	0.05	–	<0.001	0.40
DASS-21					
Anxiety	0.88	0.02	0.88	<0.001	
Stress	1.26	0.03	0.87	<0.001	

*β = unstandardized estimate; b = standardized estimate. Effect size is the proportion mediated, which is calculated by dividing the indirect effect by the total effect. Models adjusted for gender, grade, school, family economic status, and education level of caregivers. DASS-21, Depression Anxiety Stress Scales-21*.

## Discussion

In this cross-sectional study, the prevalence of EBPs in primary school children of Shanghai and Taizhou was 12.5%, with 3.3 and 2.0% children having high and very high SDQ total difficulties scores, respectively, during the post-pandemic period in China. Additionally, higher COVID-19-related knowledge and precautions were significantly associated with lower total EBPs among children after adjusting for covariates. Emotional problems in caregivers were associated with greater EBPs in children. The SEM suggested that emotional problems in caregivers partially explained the relationship between COVID-19-related knowledge and precautions and EBPs in children.

Based on this study, the prevalence of EBPs in children during the COVID-19 post-pandemic period was 12.5% in China. It was similar to a cross-sectional study conducted in Guangdong, between March 8 and March 30, 2020, which showed that the self-reported psychological disorder was 10.5% among students with an average age of 12 years (Qin et al., 2021). However, the prevalence in this study was lower than that reported in the previous studies during the COVID-19 period. In April 2020, Xie et al. reported that 22.6% of 2,330 primary school students had depressive symptoms in Hubei province during the pandemic (Xie et al., 2020). Another study, which included 3,613 students aged between 7 and 18 years from 20 provinces in mainland China, reported that 22.3% of the respondents suffered from depressive symptoms (Duan et al., 2020). The main reason for the difference was that the two studies covered Hubei province, the epicenter of the COVID-19 outbreak in China. We speculated that the survey period could be another reason. In this study, we collected the data between June 26 and July 6, 2020, when the pandemic was under control and most of the primary schools had reopened normally in China.

The results of this study provide substantial support for the association between COVID-19-related knowledge and precautions and lower psychopathology among children. This is in line with the previous findings that students who had more knowledge about COVID-19 and higher face mask-wearing frequency were less likely to experience psychological distress (Qin et al., 2021). Therefore, concerning COVID-19-related precautions, the significant inverse relationship with EBPs among children suggested that individuals who followed the recommended guidelines also had better mental health. One of the possible explanations for the protective association between pandemic-related knowledge and preventive behaviors with EBPs is that lower COVID-19-related knowledge or precautions may reflect lower intelligence of the children or family economic status, which may, in turn, be associated with poor mental health. However, the causal effects of COVID-19-related knowledge and precautions need to be verified in future prospective studies.

Our results indicate that emotional problems among caregivers, including depression, anxiety, and stress, explained 31% and 41% of the total effect of COVID-19-related knowledge and precautions, respectively, on EBPs among children during the post-pandemic period. The findings provide support for the positive relationship between emotional problems among caregivers and EBPs in children, which is consistent with previous studies (Psychogiou et al., 2017; Spinelli et al., 2020; Liang et al., 2021). A previous study indicated that as fear can be contagious, children are exceedingly sensitive to the emotional status of adults around them, who are an indispensable source of security and emotional well-being (Imran et al., 2020). Emotional problems in caregivers, such as depression, might significantly influence parent-child relations (Riley et al., 2008) and increase the risk of marital conflict and unhappiness (Cummings et al., 2005), which in turn predispose children to EBPs (Rasic et al., 2014). The pandemic caused caregivers to worry about the health of their family members and the inability to meet the demanding economic needs, especially in developing countries like China where the pandemic had badly disrupted the financial and economic activities. The consequent family economic hardship during the pandemic resulted in an increased risk of childhood psychiatric disorder (Solantaus et al., 2004). Moreover, emotional problems among caregivers are often associated with rude behaviors and difficulties in explaining the pandemic situation. Thus, children in these families may have lower personal resources to prevent negative psychological consequences (Pinquart, 2017). A steady and supportive parent-child relationship is a benefit to the positive development of the psychological well-being of young children (Schofield et al., 2013).

These findings suggest many efficient implications that should be applied to promote the well-being of children during the post-pandemic period in China. During and after the pandemic, education regarding prevention should be emphasized, and parents or caregivers should be encouraged to pay attention to what children see or hear on television or online, communicate to their children truthful and appropriate information, and teach the correct precautions including hand-washing and mask-wearing, which are important to lessen the negative effects of COVID-19 (Centers for Disease Contol and Prevention, 2020). Schools and communities have to inform children and their caregivers about the potential risk of COVID-19 and help to increase their knowledge and preventive awareness of the pandemic. Individual preventive behaviors may alleviate mental health problems in caregivers and children, and mental health intervention can improve resilience in the face of adversity among caregivers and children. It is imperative for schools and communities to not only provide the necessary health and pandemic-related information but also provide mental support to caregivers and children. The kids whose caregivers experienced psychological problems and whose families were faced with economic hardship may return to school with more intensive needs. School psychologists need to increase their level of service and consider psychoeducation to support the transition of children back into the school building and address emotional and behavioral problems. Besides, mental health providers also can assist the NGOs to organize interactive webinars with experts to help children and their caregivers cope with mental health (OECD, 2020). A previous study presented that mental disorders in children had significant consequences throughout a child's life and affected the child's development, including poor educational outcomes and a higher rate of unemployment (OECD, 2018). Thus, for children living with mental disorders, timely psychological interventions such as online counseling and cognitive behavioral therapy by trained psychologists are also critical.

Several limitations should be considered while interpreting the findings. First, causality between COVID-19-related knowledge and precautions and EBPs in children cannot be inferred from a cross-sectional study, and a longitudinal study is needed to clarify these relationships. Second, compared with a face-to-face interview, a self-report online survey was not representative of individuals who had limited access to the internet. Third, a single-item scale was used to assess COVID-19-related knowledge and precautions. Future studies could adopt a more comprehensive scale, such as a 16-item questionnaire of knowledge, attitudes, and practice toward COVID-19 used in the study of Zhong et al. (2020). Future studies could also consider measuring both self-perceived and actual knowledge to obtain a more in-depth understanding of the impact of knowledge on emotional and behavioral problems in people. Fourth, we only measured knowledge and practices toward COVID-19. It would have been better if we could also assess the attitudes of participants, such as the perceived risk of the pandemic, and evaluate their impact on the mental health of caregivers and children. Despite these limitations, this study with a large sample size offers sufficient statistical power to assess the association between COVID-19-related knowledge and precautions and EBPs in children and detect the small but plausible explanatory effect of emotional problems among caregivers on the relationship.

In conclusion, the prevalence of EBPs in primary school children was 12.5% during the post-pandemic period in China. Findings from this study suggest that levels of knowledge and preventive behaviors of COVID-19 were associated with EBPs in children, and the relationships were partially explained by the emotional problems among caregivers. Policymakers and mental health professionals should ensure that psychological interventions such as outpatient or online psychological counseling by trained psychologists are available to help children and their caregivers deal with public health crises such as the COVID-19 pandemic.

## Data Availability Statement

The raw data supporting the conclusions of this article will be made available by the authors, upon a reasonable request.

## Ethics Statement

The studies involving human participants were reviewed and approved by Institutional Review Board of School of Public Health, Fudan University. Written informed consent to participate in this study was provided by the participants' legal guardian/next of kin.

## Author Contributions

JW and YC: conceptualization, methodology, formal analysis, validation, writing-original draft, and visualization. XG and HL: conceptualization, methodology, data curation, writing review, and editing. MR: conceptualization, methodology, supervision, writing review, and editing. HW: methodology, data curation, writing review, and editing. XC: conceptualization, methodology, data curation, supervision, writing review, and editing. CF: conceptualization, methodology, supervision, funding acquisition, resources, writing review, and editing. All authors contributed to the article and approved the submitted version.

## Funding

This study was supported by the Shanghai Leading Academic Discipline Project of Public Health [Grant Nos. GWV-10.1-XK18 and GWV-10.1-XK14], and MR was supported by the Medical Research Council [Grant No. MC_UU_12019/3].

## Conflict of Interest

The authors declare that the research was conducted in the absence of any commercial or financial relationships that could be construed as a potential conflict of interest.

## Publisher's Note

All claims expressed in this article are solely those of the authors and do not necessarily represent those of their affiliated organizations, or those of the publisher, the editors and the reviewers. Any product that may be evaluated in this article, or claim that may be made by its manufacturer, is not guaranteed or endorsed by the publisher.
